# Alterations in thin filament length during postnatal skeletal muscle development and aging in mice

**DOI:** 10.3389/fphys.2014.00375

**Published:** 2014-09-29

**Authors:** David S. Gokhin, Emily A. Dubuc, Kendra Q. Lian, Luanne L. Peters, Velia M. Fowler

**Affiliations:** ^1^Department of Cell and Molecular Biology, The Scripps Research InstituteLa Jolla, CA, USA; ^2^The Jackson LaboratoryBar Harbor, ME, USA

**Keywords:** tropomodulin, actin, sarcomere, mouse, myofibril, myofilament

## Abstract

The lengths of the sarcomeric thin filaments vary in a skeletal muscle-specific manner and help specify the physiological properties of skeletal muscle. Since the extent of overlap between the thin and thick filaments determines the amount of contractile force that a sarcomere can actively produce, thin filament lengths are accurate predictors of muscle-specific sarcomere length-tension relationships and sarcomere operating length ranges. However, the striking uniformity of thin filament lengths within sarcomeres, specified during myofibril assembly, has led to the widely held assumption that thin filament lengths remain constant throughout an organism's lifespan. Here, we rigorously tested this assumption by using computational super-resolution image analysis of confocal fluorescence images to explore the effects of postnatal development and aging on thin filament length in mice. We found that thin filaments shorten in postnatal tibialis anterior (TA) and gastrocnemius muscles between postnatal days 7 and 21, consistent with the developmental program of myosin heavy chain (MHC) gene expression in this interval. By contrast, thin filament lengths in TA and extensor digitorum longus (EDL) muscles remained constant between 2 mo and 2 yr of age, while thin filament lengths in soleus muscle became shorter, suggestive of a slow-muscle-specific mechanism of thin filament destabilization associated with aging. Collectively, these data are the first to show that thin filament lengths change as part of normal skeletal muscle development and aging, motivating future investigations into the cellular and molecular mechanisms underlying thin filament adaptation across the lifespan.

## Introduction

In skeletal muscle fibers, contractile force is generated via crossbridge interactions between the myosin (thick) filaments and actin (thin) filaments in the sarcomeres arranged in series along contractile myofibrils. The capacity for a sarcomere to generate force is best described by the sliding filament theory of muscle contraction, which states that the extent of overlap between the thick and thin filaments determines the number of crossbridge interactions and, hence, the extent of active force production. The sliding filament theory can be quantified by the sarcomere length-tension relationship, which depicts a sarcomere's force output as a function of sarcomere length, and whose shape can be accurately modeled from thick and thin filament lengths (Huxley and Hanson, 1954; Edman, 1966; Gordon et al., 1966; Walker and Schrodt, 1974; Granzier et al., 1991). While thick filament lengths are constant (~1.65 μm) in all muscle types and species examined, thin filament lengths vary widely (from 0.95 to 1.40 μm) across muscle types and species, resulting in correspondingly wide variations in sarcomere length-tension relationships (reviewed in Gokhin and Fowler, 2013). The best correlate of thin filament lengths that has been identified to date is a muscle's fiber type distribution, as determined by myosin heavy chain (MHC) isoform expression, with slow-twitch muscles expressing predominantly type-I MHC (such as the soleus) having longer thin filaments, and fast-twitch muscles expressing predominantly type-II MHCs [such as the tibialis anterior (TA)] having shorter thin filament lengths in all mammalian species studied (Castillo et al., 2009; Gokhin et al., 2010, 2012). However, MHC isoform effects on thin filament length are correlative and not causative; by contrast, perturbations in thin filament pointed-end capping by tropomodulin (Tmod) can directly induce alterations in filament lengths by inhibiting actin subunit dynamics in both slow- and fast-twitch skeletal muscles (Gokhin et al., 2014).

Thin filament lengths are not fixed after their initial specification during myofibril assembly, but, rather, can exhibit remarkable plasticity in response to experimental perturbations. For example, in cardiac myocytes and *Drosophila* indirect flight muscle, thin filaments can be shortened or lengthened by increasing or decreasing, respectively, the extent of pointed-end capping by Tmod (Gregorio et al., 1995; Sussman et al., 1998; Littlefield et al., 2001; Mardahl-Dumesnil and Fowler, 2001; Tsukada et al., 2010; Bliss et al., 2014). This inverse relationship between Tmod activity and thin filament lengths appears to extend to mammalian skeletal muscle as well, as it was recently shown that proteolysis of Tmod by m-calpain can result in longer thin filaments in mouse models of Duchenne muscular dystrophy (Gokhin et al., 2014). However, it remains unclear whether changes in thin filament lengths might also be characteristic of normal muscle adaptation during an organism's lifespan (i.e., during postnatal development and aging). To address this question, we used a super-resolution computational image analysis technique (termed Distributed Deconvolution; Littlefield and Fowler, 2002) to measure thin filament lengths in skeletal muscles from mice at various stages of postnatal development, as well as from aged mice. We found that, in postnatal TA and gastrocnemius (GAS) muscles, thin filaments become shorter from postnatal days 7 to 21 (P7 to P21). This is consistent with the known developmental shift away from embryonic and neonatal MHC expression toward a fast-twitch phenotype associated with predominantly type-II MHC isoform expression (Allen and Leinwand, 2001; Agbulut et al., 2003; Gokhin et al., 2008). By contrast, in aged (2-yr-old) mice, thin filament lengths in TA and extensor digitorum longus (EDL) muscles remained constant with respect to 2-mo-old mice, while thin filament lengths in soleus muscle became shorter, suggesting muscle-specific mechanisms of length modulation. Collectively, these data identify changes in thin filament lengths as a novel feature of skeletal muscle development and aging.

## Materials and methods

### Experimental animals and tissues

Mice (*n* = 3–4 mice per time-point) were sacrificed at P7, P14, P21, 2 months after birth, or 2 years after birth. Mice sacrificed at P7, P14, and P21 were C57Bl/6J mice, while mice sacrificed at 2 months or 2 years after birth were BALB/cBy mice. Two different mouse strains were used to test whether thin filament lengths in adult skeletal muscle vary with mouse strain (i.e., C57Bl/6J at P21 vs. BALB/cBy at 2 months). Mice were sacrificed by isoflurane inhalation followed by cervical dislocation, in accordance with ethics guidelines set forth by the Institutional Animal Care and Use Committee at The Scripps Research Institute.

In experiments with P7–P21 mice, TA and GAS muscles were examined because their larger size facilitated tissue dissection and handling, and because EDL and soleus muscles are not readily distinguishable from the surrounding musculature at P7. For experiments with 2-mo- and 2-yr-old tissues, TA, EDL, and soleus muscles were examined because these muscles reflect a diversity of muscle fiber types and architectures in adult mice (Burkholder et al., 1994; Agbulut et al., 2003). GAS muscle was not analyzed in 2-mo- and 2-yr-old mice, due to its fiber type similarity with the TA and EDL (Burkholder et al., 1994).

### Immunostaining and confocal imaging

Leg muscles were stretched *in situ* via ankle joint manipulation, pinned to cork, relaxed overnight in EGTA-containing relaxing buffer, fixed overnight in 4% paraformaldehyde in relaxing buffer, dissected, embedded in Optimum Cutting Temperature compound, cryosectioned, and immunostained as previously described (Gokhin et al., 2010). Primary antibodies were as follows: mouse anti-α-actinin (EA53, 1:100; Sigma-Aldrich, St. Louis, MO) to label Z-lines; affinity-purified rabbit polyclonal anti-human Tmod1 (R1749, 3.1 μg/ml) (Gokhin et al., 2010) or rabbit polyclonal antiserum to chicken Tmod4 preadsorbed by passage through a Tmod1 Sepharose column (R3577, 1:25) (Gokhin et al., 2010) to label thin filament pointed ends; rabbit anti-nebulin M1M2M3 (NEB-1, 1:100; Myomedix, Mannheim, Germany) to label the nebulin N-terminus at the proximal/distal segment boundary of the thin filament (Gokhin and Fowler, 2013). Secondary antibodies were Alexa-488-conjugated goat anti-rabbit IgG (1:200; Life Technologies, Carlsbad, CA) and Alexa-647-conjugated goat anti-mouse IgG (1:200; Life Technologies). F-actin was stained with rhodamine-phalloidin (1:100; Life Technologies). Single optical sections were collected on a Bio-Rad Radiance 2100 laser-scanning confocal microscope mounted on a Nikon TE2000-U microscope using a 100× Plan-Apochromat oil-objective lens. Images were processed in Adobe Photoshop CS5.1, and figures were constructed in Adobe Illustrator CS5.1.

### Thin filament length measurements

Distributed Deconvolution analysis was used to measure distances of fluorescently labeled peaks of Tmod and nebulin M1M2M3 from the Z-line as well as half the breadth of the F-actin (phalloidin) signal across the Z-lines of adjacent half-sarcomeres (I-Z-I arrays), as previously described (Littlefield and Fowler, 2002; Gokhin and Fowler, 2013). Both Tmod1- and Tmod4-stained images were used for determining Tmod distances, as Tmod1 and Tmod4 are equally faithful markers of the thin filament pointed ends in all mammalian species examined (Gokhin et al., 2010, 2012). We used a distributed deconvolution plugin for ImageJ that generates the best-fit of a model intensity distribution function for a given thin filament component (Tmod, nebulin M1M2M3, or F-actin), to a background-corrected 1D myofibril fluorescence intensity profile (line-scan) of 3–5 thin filament arrays obtained for each fluorescent probe (anti-Tmod, anti-nebulin M1M2M3, or rhodamine-phalloidin). The distributed deconvolution plugin and supporting documentation are available for public download at http://www.scripps.edu/fowler/.

### Statistics

Differences between two groups were determined using Student's *t*-test. Differences between three groups were determined using One-Way ANOVA with *post-hoc* Fisher's PLSD tests. Statistical analysis was performed in Microsoft Excel. Significance was defined as *p* < 0.05.

## Results

**Figure 1:**
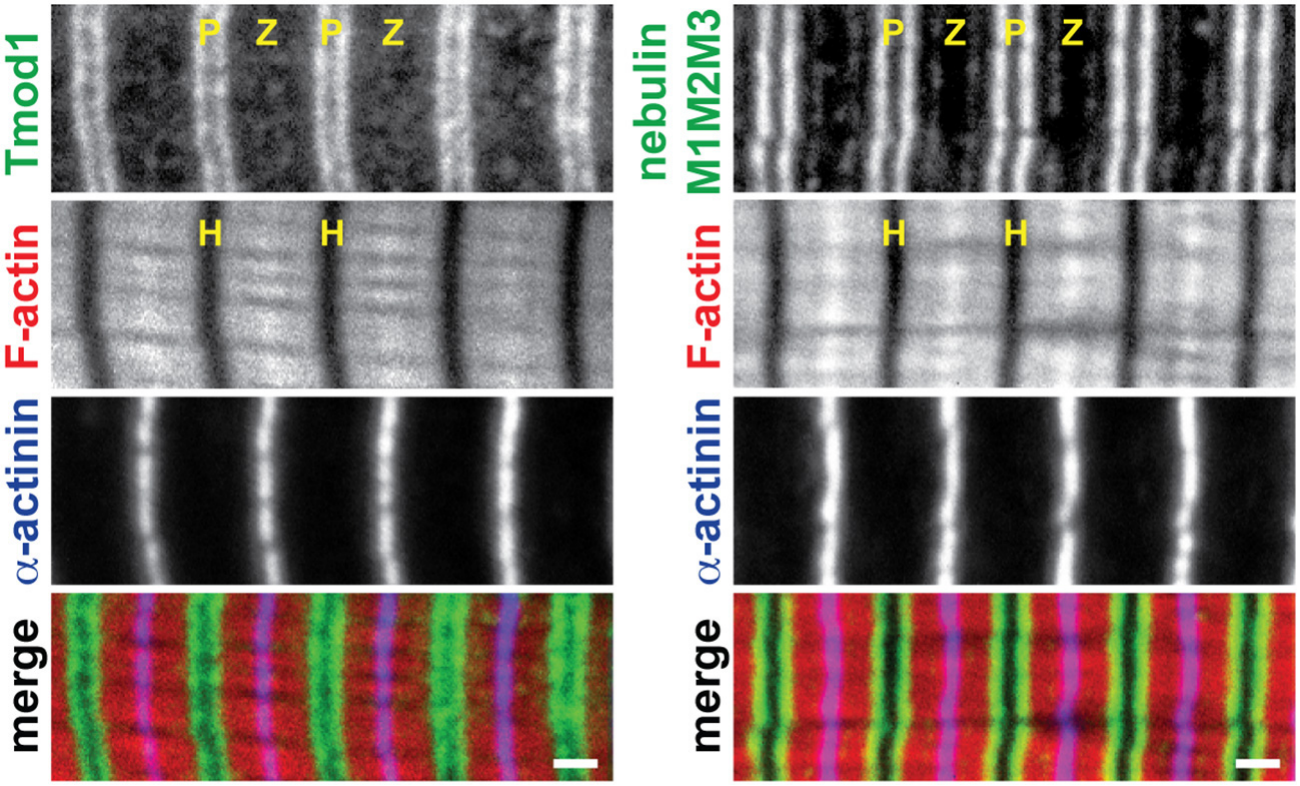
**Sample immunofluorescence images of P14 mouse muscles**. Longitudinal TA cryosections were phalloidin-stained for F-actin and immunostained for α-actinin, along with either Tmod1 or nebulin M1M2M3, and imaged by confocal microscopy. P, thin filament pointed ends; Z, Z-line; H, H-zone. Bars, 1 μm.

**Figure 2:**
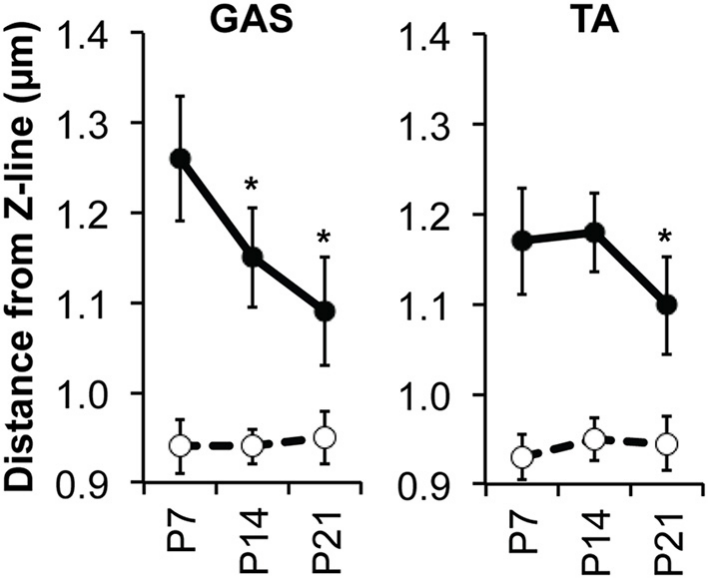
**Thin filament lengths during postnatal muscle development in mice**. Graphs depict distances of Tmod (solid circles) and nebulin M1M2M3 (open circles) from the Z-line in GAS and TA muscles from P7 to P21 mice. Tmod distance reflects the variable position of the thin filament pointed end with respect to the Z-line, while nebulin M1M2M3 distance reflects the relatively constant position of the proximal/distal segment boundary of the thin filament with respect to the Z-line (Gokhin and Fowler, 2013). Tmod distances reflect pooled Tmod1 and Tmod4 distances. Error bars reflect mean ± *SD* for *n* ≥ 100 myofibrils/time-point for Tmod, *n* ≥ 50 myofibrils/time-point for nebulin M1M2M3. ^*^p < 0.01 with respect to the previous time-point.

To examine changes in thin filament length during postnatal skeletal muscle development, we used confocal fluorescence microscopy and a super-resolution computational image analysis approach (Distributed Deconvolution; Littlefield and Fowler, 2002) to measure thin filament lengths in mouse skeletal muscles from P7 to P21. Organized myofibrils with well-regulated thin filament lengths were evident in all muscles examined at all postnatal time-points, as evidenced by striated Tmod1, Tmod4, nebulin M1M2M3, F-actin, and α-actinin staining patterns (sample images from P14 muscle shown Figure 1). In the postnatal GAS, thin filament lengths determined by Tmod1 localization steadily decreased from 1.26 to 1.09 μm from P7 to P21 (Figure 2; Table 1). In the postnatal TA, thin filament lengths stayed constant at ~1.17 μm from P7 to P14, but decreased to 1.10 μm by P21 (Figure 2; Table 1). Thin filament lengths determined by F-actin breadth from the Z-line to the H-zone paralleled those determined by Tmod1 localization in both neonatal GAS and TA muscles at all time-points (Table 1). Nebulin M1M2M3 distances from the Z-line remained constant at ~0.94 μm in both neonatal GAS and TA (Figure 2; Table 1), consistent with the nebulin-coated proximal segment of the thin filament having a fixed length, regardless of the length of the nebulin-free distal segment (Gokhin and Fowler, 2013).

**Table 1:** **Thin filament lengths determined by Distributed Deconvolution analysis of fluorescence images**.

		**Tmod**	**Phalloidin**	**Nebulin M1M2M3**
GAS	P7	1.26 ± 0.07	1.16 ± 0.03	0.94 ± 0.03
	P14	1.15 ± 0.06^*^	1.06 ± 0.02^*^	0.94 ± 0.02
	P21	1.09 ± 0.06^*^	0.98 ± 0.02^*^	0.95 ± 0.03
TA	P7	1.17 ± 0.06	1.10 ± 0.02	0.93 ± 0.02
	P14	1.18 ± 0.04	1.09 ± 0.02	0.95 ± 0.02
	P21	1.10 ± 0.07^*^	1.01 ± 0.04^*^	0.95 ± 0.03
	2 mo	1.11 ± 0.06	1.03 ± 0.04	0.93 ± 0.03
	2 yr	1.08 ± 0.07	1.00 ± 0.04	0.94 ± 0.03
EDL	2 mo	1.07 ± 0.07	1.00 ± 0.06	0.94 ± 0.02
	2 yr	1.09 ± 0.06	1.03 ± 0.05	0.94 ± 0.02
Soleus	2 mo	1.19 ± 0.05	1.14 ± 0.11	0.96 ± 0.03
	2 yr	1.11 ± 0.02^*^	1.06 ± 0.05^*^	0.95 ± 0.03

Length values (all in μm) correspond to the breadth of phalloidin staining and the distances of Tmod and nebulin M1M2M3 from the Z-line. Data are shown mean ± SD for n ≥ 100 myofibrils/time-point for Tmod and phalloidin, n ≥ 50 myofibrils/time-point for nebulin M1M2M3.

* *p < 0.01 with respect to the previous time-point*.

**Figure 3:**
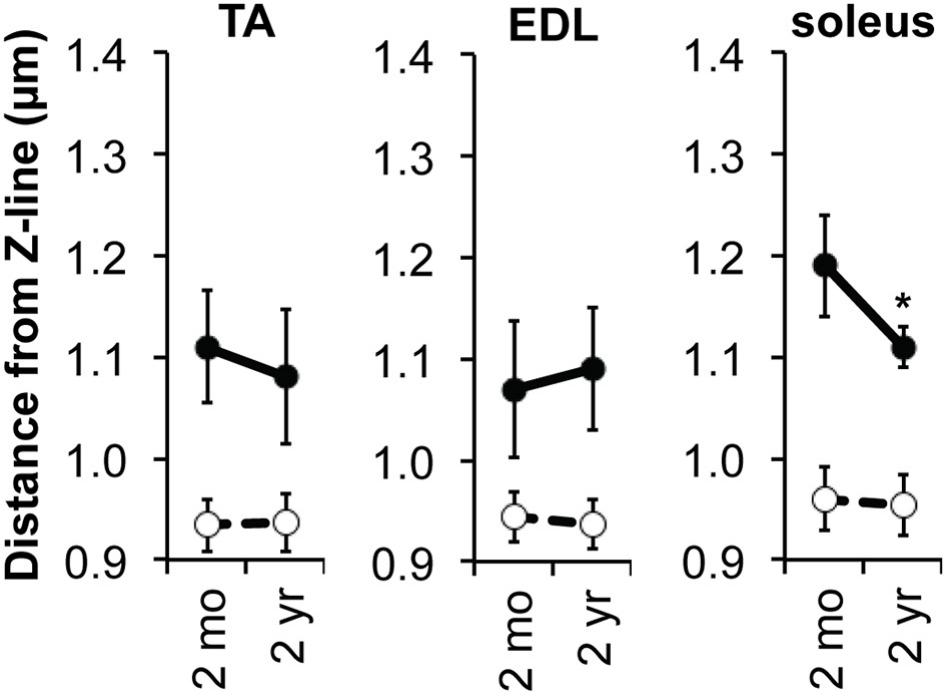
**Thin filament lengths in aged mouse muscles**. Graphs depict distances of Tmod (solid circles) and nebulin M1M2M3 (open circles) from the Z-line in TA, EDL, and soleus muscles from 2-mo-old to 2-yr-old mice. Tmod distance reflects the variable position of the thin filament pointed end with respect to the Z-line, indicative of variations in length, while nebulin M1M2M3 distance reflects the relatively constant position of the proximal/distal segment boundary of the thin filament with respect to the Z-line (Gokhin and Fowler, 2013). Tmod distances reflect pooled Tmod1 and Tmod4 distances. Error bars reflect mean ± *SD* for *n* ≥ 100 myofibrils/time-point for Tmod, *n* ≥ 50 myofibrils/time-point for nebulin M1M2M3. ^*^p < 0.01 with respect to the previous time-point.

Next, to examine changes in thin filament length during skeletal muscle aging, we further used confocal fluorescence microscopy and Distributed Deconvolution analysis to compare thin filament lengths in 2-mo-old vs. 2-yr-old mice. Aged muscles showed no evidence of gross myofibril disorganization or deterioration, as evidenced by striated Tmod1, nebulin M1M2M3, F-actin, and α-actinin staining (data not shown). In the TA and EDL, no statistically significant differences in thin filament lengths were observed between 2-mo-old vs. 2-yr-old muscles (~1.11 μm in both muscles at both time points) (Figure 3; Table 1). However, in the soleus, thin filament length decreased from ~1.20 μm in 2-mo-old muscles to ~1.11 μm in 2-yr-old muscles (Figure 3; Table 1). As observed with our measurements of thin filament lengths in neonatal muscles, thin filament lengths determined by F-actin breadth from the Z-line to the H-zone paralleled those determined by Tmod1 localization (Table 1), and nebulin M1M2M3 distances from the Z-line remained constant at ~0.95 μm (Figure 3; Table 1). Moreover, thin filament lengths in the TA muscles of 2-mo-old BALB/cBy mice were identical to thin filament lengths in the TA muscles of P21 C57Bl/6J mice (Table 1; also compare Figure 2 and Figure 3), indicating that thin filament lengths do not change between P21 and 2 months of age, and that lengths are not mouse strain-dependent. Thin filament lengths in adult mice in these strains also agree with lengths previously reported in adult mixed-background FVB/N:129/SvJ:C57Bl/6 mice (Gokhin et al., 2010).

## Discussion

This study provides the first evidence that skeletal muscle thin filaments undergo adaptive remodeling to alter their lengths during the mouse lifespan, implying age-dependent alterations in sarcomere length-tension relationships and optimum sarcomere length ranges. What molecular mechanisms might drive these changes in thin filament lengths? When examining neonatal muscles, we observed thin filament shortening between P7 and P21 in both the TA and GAS (Figure 2). These changes are mostly associated with developmental transitions away from expression of embryonic and neonatal MHC isoforms and toward expression of adult type-II MHC isoforms (Allen and Leinwand, 2001; Agbulut et al., 2003; Gokhin et al., 2008). The presence of type-II MHC isoforms is, in turn, associated with shorter thin filament lengths (Castillo et al., 2009; Gokhin et al., 2010, 2012). This suggests a model in which immature mouse muscles, expressing predominantly embryonic and neonatal MHCs, are all initially specified with “generic” thin filament lengths during the terminal stages of myofibril assembly, despite these muscles' functional differences in adult mice (Allen and Leinwand, 2001; Agbulut et al., 2003; Gokhin et al., 2008). Then, as the various muscles adopt their characteristic use profiles and MHC isoform distributions during postnatal acquisition of adult movement patterns, thin filament lengths may gradually diversify, ultimately leading to the multitude of muscle-specific thin filament lengths observed in adult mice (i.e., longer thin filaments characteristic of slower fiber types and shorter filaments characteristic of faster fiber types) (Gokhin et al., 2010, 2014). Simultaneously, postnatal changes in tropomyosin and troponin isoform expression (Amphlett et al., 1976; Briggs et al., 1990) may also be involved in regulating thin filament lengths during postnatal skeletal muscle development. Evidence supporting this possibility is the fact that a nemaline myopathy-causing tropomyosin mutation can cause markedly shorter thin filaments *in vivo* (Ochala et al., 2012), and that both tropomyosin and troponin have been shown to enhance actin filament pointed-end stability *in vitro* (Broschat et al., 1989; Broschat, 1990; Weigt et al., 1990).

When comparing 2-mo-old and 2-yr-old muscles, we observed no statistically significant changes in thin filament length in the TA or EDL, but a marked decrease in thin filament length was observed in the 2-yr-old soleus (Figure 3). The fact that thin filament shortening during aging is restricted to the soleus muscle implies a mechanism specific to heavily recruited, slow-twitch muscles. Such a mechanism most likely does not involve shifts toward expression of type-II MHC isoforms during aging, unlike the MHC isoform shifts described above for postnatal muscle development, because studies of rat soleus have shown continued enrichment of type-I MHC with decreases in type-II MHC during aging (Butler-Browne and Whalen, 1984; Larsson et al., 1995). Indeed, an increase in type-I MHC with concomitant thin filament shortening is not consistent with a causative effect of MHC isoforms in regulating thin filament lengths. Other molecules whose alterations might directly induce slow-muscle-specific thin filament shortening in aging are tropomyosin (an actin side-binding and stabilizing protein), whose α isoforms are decreased in aged muscle (Gelfi et al., 2006; O'Connell et al., 2007), as well as ADF/cofilin (an actin-severing protein) and profilin (an actin monomer-sequestering protein), whose levels are increased in degenerating muscle (Nagaoka et al., 1996). Moreover, slow-muscle-specific changes in thin filament protein phosphorylation, glycosylation, or other posttranslational modifications might also contribute to slow-muscle-specific thin filament shortening in aging. Understanding the contributions and interplay of these molecules and pathways to thin filament shortening in aging requires further studies.

An alternative mechanism for slow-muscle-specific thin filament shortening in aging involves preferential induction of proteolytic pathways in slow-twitch muscles (Sultan et al., 2001), leading to breakdown of thin filament-associated proteins and reduced F-actin stability. Such proteolytic pathways might be specifically induced in old muscle, or might be ongoing in heavily recruited muscles and begin in mid-life. A number of proteolytic pathways have been implicated in skeletal muscle aging and aging-related sarcopenia, including activation of intracellular proteases (calpains and caspases), the ubiquitin-proteasome system, the autophagy-lysosome system, and reactive oxygen species (for reviews, see Fulle et al., 2004; Dargelos et al., 2008; Ryall et al., 2008; Combaret et al., 2009). Activation of m-calpain can lead to Tmod proteolysis that is more widespread in slow muscle in mouse models of Duchenne muscular dystrophy, but this mechanism results in actin subunit addition onto pointed ends and resultant thin filament elongation, and not thin filament shortening (Gokhin et al., 2014). More likely targets whose proteolysis could lead to shorter thin filaments are actin filament side-binding proteins such as nebulin and tropomyosin, which stabilize thin filaments to prevent aberrant thin filament shortening (Bang et al., 2006; Ottenheijm et al., 2009; Pappas et al., 2010; Ochala et al., 2012). Ongoing work seeks to understand the regulatory framework governing the interplay between aging and pathology of skeletal muscle, alterations in thin filament proteins, and regulation of thin filament lengths.

### Conflict of interest statement

The authors declare that the research was conducted in the absence of any commercial or financial relationships that could be construed as a potential conflict of interest.
